# Alterations of actin cytoskeleton and arterial protein level in patients with obstructive jaundice

**DOI:** 10.1590/1678-4685-GMB-2021-0419

**Published:** 2022-09-12

**Authors:** Hong-Qian Wang, Xiao-Yan Meng, Jin-Min Zhang, Jia-Ying Chen, Bao-Hua Zhang, Fei-Xiang Wu

**Affiliations:** 1 Naval Medical University, Shanghai Eastern Hepatobiliary Surgery Hospital, Department of Critical Care Medicine, Shanghai, China.; 2 First Medical University, Shandong Provincial Hospital Affiliated to Shandong Jinan, Department of Anesthesiology, Shandong, China.; 3 Naval Medical University, Shanghai Eastern Hepatobiliary Surgery Hospital, Department of Anesthesiology, Shanghai, China.; 4 Naval Medical University, The Eastern Hepatobiliary Surgery Hospital, Department of Biliary Tract Surgery, Shanghai, China.

**Keywords:** Vascular hypo-reactivity, obstructive jaundice, proteomics, artery, hemodynamic changes

## Abstract

Vascular hypo-responsiveness to vasopressors in patients with obstructive jaundice (OJ) is a common anesthetic event, which leads to perioperative complications and increased mortality. The cause of this clinical issue remains unclear. In this study, we estimated the actin cytoskeleton and arterial protein level in the artery of OJ patients by proteomic analysis. Ten patients with OJ due to bile duct diseases or pancreatic head carcinoma were enrolled, while another ten non-jaundice patients with chronic cholecystitis or liver hemangioma as the control group. Vascular reactivity to noradrenaline was measured before anesthesia on the day of surgery. Artery samples in adjacent tissues of removed tumor were collected and evaluated by 2-dimensional electrophoresis. Proteins with differential expression were detected by MALDI-TOF mass spectrometry with immunoblot confirmation. The results confirmed the phenomenon of vascular hypo-reactivity in OJ patients as suppressed aortic response to noradrenaline were existed in these patients. We also found that actin cytoskeleton and several actin-binding proteins were up- or down-regulated in the artery of OJ patients. These proteins changed in OJ patents might be the basic mechanism of vascular hypo-reactivity, further studies to uncover the role of these proteins in OJ is critical for clinical treatment of these patients.

## Introduction

Obstructive jaundice (OJ) is a common clinical symptom which could attributed to gallstones, malignant tumors and other ailments. Most OJ patients need surgery for treating of primary disease. However, the incidences of perioperative complications and mortality are higher in OJ cases compared with non-jaundice patients (Sewnath *et al.*, 2002). This is mainly due to perioperative hypotension, acute renal failure and other complications caused by endotoxemia (Binah *et al.*, 1985; Green and Better, 1995). What’s more, hypotension is a highly rated and prominent clinical mark for anesthesia in OJ during operation (Rege, 1995). 

Our previous study found that impaired arterial baroreflex sensitivity may play a role in enhancing susceptibility to perioperative complications encountered in OJ patients during anesthesia (Song *et al.*, 2009), consistent with other studies (Finberg *et al.*, 1981; Bomzon *et al*., 1986). However, the molecular mechanisms behind vascular function change in OJ remain unclear, and morbidity and overall mortality of low blood pressure, renal failure or multiple organ dysfunction syndrome cannot be improved until suitable treatment for vascular injury in jaundice is developed. 

In the current work, changes in the artery of OJ patients were evaluated. This study aimed to assess the arterial proteome for differentially expressed proteins in various databases, to provide insights into the molecular mechanisms underpinning vascular hypo-responsiveness in patients with OJ.

## Material and Methods

### Patients

Eligibility criteria were based on our previous work (Song *et al*., 2009). This trial had approval from the Shanghai ethics committee (Register Number: ACTRN12611000980932). All subjects participating in the current trial provided signed informed consent. The trial was registered prior to patient enrollment in ANZCTR (Australia New Zealand Clinical Trials Registry; Research Register Number, U1111-1124-2612; Principal investigator, Feixiang Wu; Registered Data, 09/2011). Ten patients with obstructive jaundice due to bile duct or pancreatic head carcinoma were enrolled, while ten non-jaundice patients with chronic cholecystitis or liver hemangioma cases were included as the control group. All patients with American Society of Anesthesiologists physical statuses I and II, respectively, and were 40-70 years old. All participants were scheduled for elective surgery for the underlying diseases from June 2011 to December 2011 in Eastern Hepatobiliary surgical hospital, Shanghai, China.

Exclusion criteria comprised: (1) age>70 years or <20 years; (2) severe obesity (body mass index >30 kg/m^2^); (3) previous cardiovascular, pulmonary, or kidney disease; (4) hepatic encephalopathy, psychiatric disease, or neuropathology; (5) acid-base balance alteration, blood electrolyte imbalance, diabetes, sepsis, or overt weight loss resulting from cancer. 

### Vascular reactivity measurement

Vascular reactivity was measured prior to anesthesia on the day of surgery. Upon arrival to the operating room following fasting (8 to 10 h), electrocardiography monitoring (lead II), finger pulse oximetry and non-invasive blood pressure assessment were carried out. Under local anesthesia, the left radial artery was punctured and catheterized, and connected via a sensor to a waveform analyzer (Vigileo system, Edwards life sciences, USA). Parameters were set with no adjustment, for continuous monitoring of cardiac output (CO), cardiac output index (CI), each-volume (SV), stroke volume index (SVI) and stroke volume variation (SVV). After puncturing the right internal jugular vein and indwelling a three-cavity central venous catheter under local anesthesia, central venous pressure (CVP) was simultaneously recorded. Body circulation resistance (SVR) and body circulation resistance index (SVRI) were calculated by inputting the obtained CVP values. Recorded values were averaged from three separate measurements. Ringer lactate solution was continuously instilled at 2 ml/kg/h. The patients were placed in the supine position for 20 minutes or more after entering the surgical room, and vascular reactivity was observed upon tension relief. After recording baseline values for mean arterial pressure (MAP), HR, CO, CI, SVR and SVRI, continuous infusion of increasing concentrations of noradrenaline for 5 min (30 and 60 ng.kg^-1^min^-1^) was performed, with a 20-minute interval between the two concentrations to allow recovery. Systolic blood pressure did not exceed 160 mmHg at the maximum dose. Stable for 3 minutes during each administration, systolic blood pressure and the heart rate returned to pre-test levels, fluctuating up and down within 5%.

### Preparation of total protein extracts

Artery samples near or surrounding the removed tissues were collected. About 100 mg of these samples were washed with cold PBS buffer three times. After PBS removal, 1 ml of lysis buffer (9.5 M Urea, 65 mM DTT, 4% CHAPS and 0.2% IPG buffer, mixed with enzyme inhibitors at 50:1 v/v) was added. The samples were then homogenized with a Dounce homogenizer after vertexing. Next, ultrasonication of the specimens was performed 5 times at 15-second intervals on ice (80 W, 10 seconds). Supernatants were collected and stored at -80 ℃ after centrifugation (12,000 g, 4 ℃) for 45 minutes. Quantitative analysis of total protein in artery samples was performed with Bio-Rad protein assay reagents.

### Two-dimensional electrophoresis (2-DE)

Proteins (100 μg) were firstly resolved by isoelectrofocusing (IEF) with gel strips measuring pH values between 3 and 10 based on a non-linear gradient (IPG strip, 18 cm; GE Healthcare, Waukesha, WI, USA). Strip rehydration was carried out in a Protein IEF Cell (GE Healthcare) in the passive (4 h) and active (12 h) modes at room temperature. Simultaneous IEF was carried out on an Ettan IPGphor Isoelectric Focusing system as follows: (a) 30 V for 12 h; (b) voltage gradient to 500 V in 1 h; (c) gradient to 1000 V in 1 h; (d) gradient to 8000 V in 8 h; (e) 500 V for 4 h. Next, IPG strips were placed onto vertical 12.5% polyacrylamide gels in an Ettan-DALT Six system (GE Healthcare). Second-dimension SDS-PAGE was performed on a Hofer SE 600 system (GE Healthcare) at 5 W/gel for 30 min followed by 180 W for 4 h. Protein spots were detected after Coomassie blue dyes on an UMax Powerlook 2110XL scanner (GE Healthcare).

### Trypsin digestion and gel extraction

Spots of interest were excised from Coomassie Brilliant Blue stained gels after 2 Milli-Q water (15 min) and 4 NH4HCO3 (25 mM in 50% acetonitrile; 30 minutes) washes. After washing, the gel pieces were submitted to acetonitrile (50 mL) dehydration until opacity and further placed at 60º C to total dryness; rehydration was carried out in 50 mM NH4HCO3. Next, the samples were digested with trypsin (12.5 ng/mL; Promega, Madison, USA) in 10-mL reactions. The samples were chilled on ice for 45 min followed by addition of 25 mM NH4HCO3 and overnight incubation (37º C). After centrifugation, supernatants were submitted to 3 extractions with 50% acetonitrile containing 0.1% trifluoroacetic acid. The resulting mixtures were vacuum-dried. In MALDI-TOF/TOF, peptide elution was performed with 0.7 mL α-cyano-4-hydroxy-cinnamic acid (Sigma, St. Louis, MO, USA) in 0.1% trifluoroacetic acid/50% acetonitrile.

### Mass spectrometry

A 5800 MALDI TOF/TOF analyzer (Applied Biosystem, Framingham, MA, USA) was employed for assessments. Mass spectrum (m/z 800-4000) acquisition was in the positive ion reflector mode. The top 20 ions in terms of intensity were submitted to MS/MS sequencing in the 2 kV mode. The NCBI protein database was queried for matched MS/MS spectra by Matrix Science (http://www.matrixscience.com) for the identification of proteins: taxonomy, Green Plants; peptide tolerance, 1.2 Da; MS/MS tolerance, 0.6 Da; 1 incomplete cleavage allowed; modification, methionine oxidation. Based on MASCOT probability assessment (P<0.05), significant hits were used for protein identification. The identified proteins were examined for sub-cellular localization and function with TargetP (http://www.cbs.dtu.dk/services/TargetP/) or PSORT (http://psort.hgc.jp/). Further, potentially related pathways and biochemical reactions were queried in Uniprot (http://www.uniprot.org/) and Genome-Net (http://www.genome.ad.jp/kegg/). The STRING toll (https://string-db.org/) was used to make the protein-protein interaction networks and analysis the biological process.

### Western blot

Aorta segment lysis was performed with chilled lysis buffer in presence of protease inhibitors. Lysate centrifugation was carried out at 12,000 rpm for 15 min at 4 °C. Total protein in the supernatant was quantitated with a BSA assay kit (P0006, Beyotime, Jiangsu, China). Protein separation was performed by 10% sodium dodecyl sulfate polyacrylamide gel electrophoresis (SDS-PAGE), and protein bands were electro-transferred onto polyvinylidene difluoride (PVDF) membranes. The samples were incubated overnight at 4 °C with anti-tropomyosin β, anti-transgelin, anti-annexin, anti-gelsolin, anti-HSP-27, anti-cofilin-1 and anti-GAPDH (1:1000; Abcam, Cambridge, UK) primary antibodies. Further incubation was performed with goat anti-mouse or anti-rabbit secondary antibodies (1:10,000; Santa Cruz Biotechnology, USA) for 1h in ambient conditions. Development was carried out with the BeyoECL kit (Beyotime, China) and a Tanon 5200 system.

### Statistical analysis

Spot intensities for differentially expressed proteins in D gels were obtained from three independent experiments. One-way analysis of variance and Duncan’s multiple range test were employed for comparisons. P<0.05 at two tails was deemed statistically significant.

## Results

### General conditions

Age, gender distribution, height, weight and BSA were comparable in both groups. Serum total bilirubin and bile acid amounts were markedly elevated in OJ patients compared with controls (all P<0.01; Table 1).

**Table 1 -:** Demographic data.

	Control (N=10)	Obstructive Jaundice (N=9)	P value
Sex, M/F	6/4	5/4	0.84
Age, yr	53.5±3.63	55.8±3.38	0.17
Height, cm	164.8 ± 9.16	161.7 ± 8.27	0.44
Weight, kg	61.76 ± 11.59	59.71 ± 10.95	0.68
BSA, m^2^	1.68 ± 0.20	1.63 ± 0.19	0.58
Total bilirubin, µM	12.2 ± 0.80	162.6 ± 28.41*	<0.001
Bile acids, µM	6.6 ± 0.45	81.5 ± 13.89*	<0.001

BSA: body surface area. Data are presented as Mean ± SD.

### Vascular reactivity

**Figure 1 -:**
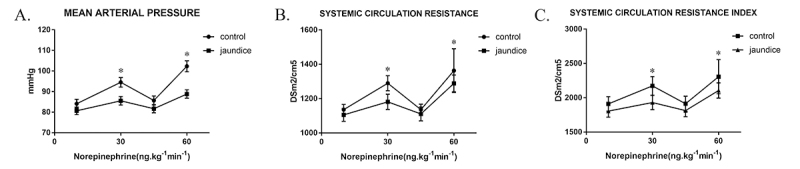
Vascular reactivity after agent administration. A. Measured MAP values in OJ and non-OJ patients (P=0.013, two-way ANOVA with post hoc Bonferroni correction, N=10 in Jaundice group while 9 in Control). B. Measured SVR in OJ and non-OJ patients (P=0.08, two-way ANOVA with post hoc Bonferroni correction, N=10 in Jaundice group while 9 in Control). C. Measured SVRI in OJ and non-OJ patients (P=0.11, two-way ANOVA with post hoc Bonferroni correction, N=10 in Jaundice group while 9 in Control).

Baseline values for hemodynamic data such as MAP, heart rate (HR), CVP, CO, CI, SVR and SVRI were comparable in both groups (Figure 1). Increases in dose-related MAP, SVR and SVRI were observed in both groups compared with baseline values (Figure 1). After continuous infusion of 30 ng.kg^-1^min^-1^ norepinephrine for 5 min, MAP increase in jaundice patients (from 80.7±5.9 mmHg to 85.5±6.6 mmHg) was lower than that of the non-jaundice group (from 84.1±6.7 mmHg to 94.5±7.4 mmHg, p<0.05). Similarly, after continuous pumping of 60 ng.kg^-1^min^-1^ norepinephrine for 5 min, MAP increase in patients with jaundice was significantly lower than of the non-jaundice group (from 81.7±6.3 mmHg to 88.8±6.5 mmHg and from 85.7±6.9 mmHg to 102.3±8.4 mmHg, respectively; p<0.05). 

After continuous pumping of norepinephrine, SVR increases were less pronounced in the jaundice group (from 1105.45±119.01 dyn.s.cm^-5^m^2^ to 1181.09±141.90 dyn.s.cm^-5^m^2^ at a dose of 30 ng.kg^-1^min^-1^ vs. from 1110.86±127.86 dyn.s.cm^-5^m^2^ to 1288.62±154.71 dyn.s.cm^-5^m^2^ at 60 ng.kg^-1^min^-1^, p<0.05) in comparison with the non-jaundice group (from 1135.75±95.80 dyn.s.cm^-5^m^2^ to 1289.96±139.04 dyn.s.cm^-5^m^2^ at a dose of 30 ng.kg^-1^min^-1^ vs. from 1135.75±101.47 dyn.s.cm^-5^m^2^ to 1472.01±155.63 dyn.s.cm^-5^m^2^ at 60 ng.kg^-1^min^-1^, p<0.05). Similarly, SVRI increases were less pronounced in the jaundice group (from 1805.29±276.31 dyn.s.cm^-5^m^2^ to 1930.89±329.48 dyn.s.cm^-5^m^2^ at a dose of 30 ng.kg^-1^min^-1^ vs. from 1813.52±281.06 dyn.s.cm^-5^m^2^ to 2105.65±352.91 dyn.s.cm^-5^m^2^ at 60 ng.kg^-1^min^-1^, p<0.05) in comparison with the non-jaundice group (from 1911.25±333.43 dyn.s.cm^-5^m^2^ to 2172.80±426.08 dyn.s.cm^-5^m^2^ at a dose of 30 ng.kg^-1^min^-1^ vs. 1912.70±349.62 dyn.s.cm^-5^m^2^ to 2477.27±463.41 dyn.s.cm^-5^m^2^ at 60 ng.kg^-1^min^-1^, p<0.05). There were no statistical differences in CO and CI changes between the patient and control groups.

### Proteomics findings

**Figure 2 -:**
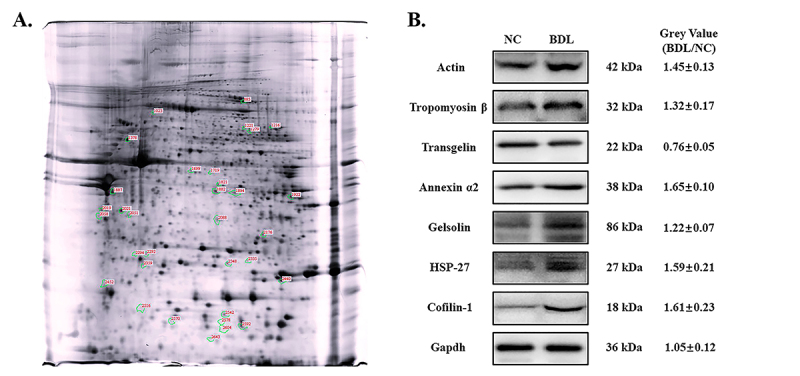
Differentially expressed protein spots in artery of obstructive jaundice patients. A. Representative analytical proteome map of obstructive jaundice. B. Western blot analysis for seven actin or actin-related proteins identified by DIGE analysis.

In order to determine quantitative expression differences in proteins between obstructive jaundice and non-jaundice patients, the proteomic profiles of both groups were analyzed. In this comparison, thirty-three differentially expressed spots were identified by 2-DE image analysis (Figure 2A). 

### Confirmation of proteomics findings 

We further used Western blot to confirm seven actin or actin related proteins in the cytoskeleton signaling pathway. As shown in Figure 2B, the expression levels of actin, tropomyosin β, transgelin, annexin, gelsolin, HSP-27 and cofilin-1 were in accordance with proteomics data.

### Bioinformatics findings

A total of 24 proteins with differential expression between obstructive jaundice and non-jaundice patients were identified by MS/MS. They included 17 upregulated and 7 downregulated proteins (Table 2).

**Table 2 -:** Identification results of proteins expressed in obstructive jaundice vs. non-jaundice patients.

protein name	Accession NO.	Fold change (T/N)	Functional annotation^#^	Gene	Cellular component
Actin, aortic smooth muscle	P62736	1.56782	Highly conserved proteins that are involved in various types of cell motility and are ubiquitously expressed in all eukaryotic cells.	ACTA2	Cytoplasm, cytoskeleton.
Actin, gamma-enteric smooth muscle	P63267	1.47153	Highly conserved proteins that are involved in various types of cell motility and are ubiquitously expressed in all eukaryotic cells.	ACTG2	Cytoplasm, cytoskeleton.
Actin, cytoplasmic 2	P63261	1.84219	Highly conserved proteins that are involved in various types of cell motility and are ubiquitously expressed in all eukaryotic cells.	ACTG1	Cytoplasm, cytoskeleton.
Tropomyosin beta chain	P07951	1.29052	Binds to actin filaments in muscle regulates vertebrate striated muscle contraction; regulates smooth muscle contraction.	TPM2	Cytoplasm, cytoskeleton.
Transgelin	Q01995	-1.23158	Involves in calcium interactions and contractile properties of the cell that may contribute to replicative senescence	TAGLN	Cytoplasm.
Vimentin	P08670	2.15428	Class-III intermediate filaments found in various non-epithelial cells, and is attached to the nucleus, endoplasmic reticulum, and mitochondria.	VIM	Cell membrane, cytoplasm. cytoskeleton, intermediate filament, membrane, nucleus.
Annexin A2	P07355	1.51842	May be involves in heat-stress response.	ANXA2	Basement membrane, extracellular matrix, secreted.
Annexin A5	P08758	1.36364	Involves in the blood coagulation cascade.	ANXA5	Cytosol, extracellular region or secreted, plasma membrane.
Gelsolin	P06396	1.20617	Prevents monomer exchange (end-blocking or capping), promote the assembly of monomers into filaments (nucleation) as well as sever filaments already formed.	GSN	Amyloid, cytoplasm, cytoskeleton, secreted.
Cofilin-1	P23528	1.35534	Binds to F-actin and exhibits pH-sensitive F-actin depolymerizing activity, regulates cell morphology and cytoskeletal organization in epithelial cells	CFL1	Cell membrane, cell projection, cytoplasm, cytoskeleton, membrane, nucleus.
LIM and SH3 domain protein 1	Q14847	-1.68030	Plays an important role in the regulation of dynamic actin-based, cytoskeletal activities, regulates actin-associated ion transport activities.	LASP1	Cytoplasm, cytoskeleton.
Sorbin and SH3 domain-containing protein 2	O94875	-1.41926	Adapter protein that plays a role in the assembling of signaling complexes; may participates in the regulation of pancreatic cell adhesion; isoform 6 increases water and sodium absorption in the intestine and gall-bladder.	SORBS2	Cell junction, cell membrane, cell projection, cytoplasm, membrane.
Keratin, type I cytoskeletal 9	P35527	1.20084	May involves in the mature palmar and plantar skin tissue.	KRT9	Intermediate filament, keratin.
Prelamin-A/C	P02545	-1.30005	Participates in nuclear assembly, chromatin organization, nuclear membrane and telomere dynamics.	LMNA	Intermediate filament, nucleus.
Delta(3,5)-Delta(2,4)-dienoyl-CoA isomerase	Q13011	-1.45960	Isomerization of 3-trans,5-cis-dienoyl-CoA to 2-trans,4-trans-dienoyl-CoA.	ECH1	Mitochondrion, peroxisome.
Galactokinase	P51570	1.86176	Catalyzes the transfer of a phosphate from ATP to alpha-D-galactose and participates in the first committed step in the catabolism of galactose.	GALK1	Cytosol, extracellular region or secreted, extracellular exosome Source.
Pyruvate kinase PKM	P14618	1.20084	Generates ATP; regulates transcription; tumor cell proliferation and survival.	PKM	Nucleus, cytoplasm and cytosol.
Protein AMBP	P02760	1.42851	1. Alpha-1-microglobulin: removes and protects against harmful oxidants and repairs macromolecules in intra- and extra-vascular spaces; intravascularly, plays a regulatory role in red cell homeostasis. 2. Inter-alpha-trypsin inhibitor light chain: extracellular space remodeling and cell adhesion.	AMBP	Nucleus, extracellular region or secreted, plasma membrane, cell membrane, cytoplasm and cytosol, mitochondrion, peripheral membrane protein.
Glutathione S-transferase P	P09211	1.46583	Involves in the formation of glutathione conjugates of both prostaglandin A2 (PGA2) and prostaglandin J2 (PGJ2).	GSTP1	Cytoplasm, mitochondrion, nucleus.
Fibrinogen beta chain	P02675	-1.36779	Fibrin has a major function in hemostasis as one of the primary components of blood clots; involved in the early stages of wound repair; associated with infection and facilitate the antibacterial immune response.	FGB	Secreted.
Biglycan	P21810	-1.29570	May be involved in collagen fiber assembly.	BGN	Extracellular matrix, secreted.
Heat shock protein beta-1	P04792	1.34520	A molecular chaperone probably maintaining denatured proteins in a folding-competent state, and involves in stress resistance and actin organization.	HSPB1	Cytoplasm, cytoskeleton, nucleus.
Polymerase I and transcript release factor	Q6NZI2	1.75672	Involves in caveolae formation and organization in all tissues.	CAVIN1	Cell membrane, cytoplasm, endoplasmic reticulum, microsome, mitochondrion, nucleus.
Ras suppressor protein 1	Q15404	-2.15990	Potentially plays a role in the Ras signal transduction pathway.	RSU1	Cytosol, extracellular region or secreted, etc.

Functional annotations for all proteins are based on Uniport (https://www.uniprot.org/).

**Figure 3 -:**
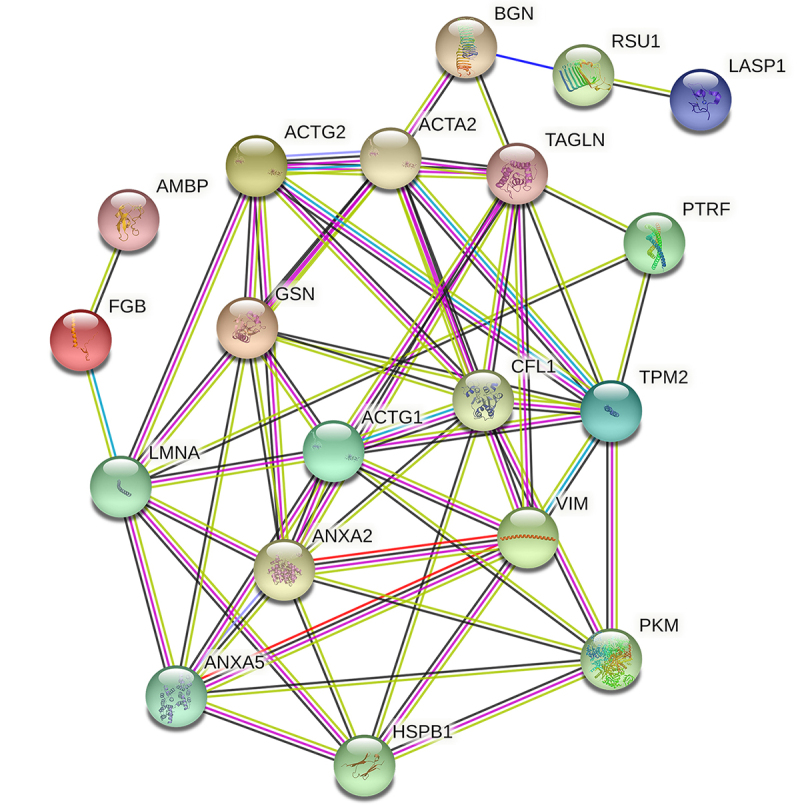
Protein-protein interaction maps of the differentially expressed protein spots in artery of obstructive jaundice patients. The STRING toll (https://cn.string-db.org) was used to make the networks and analysis the biological process. The line color indicates the type of the interaction evidence. Network nodes represent proteins splice isoforms or post-translational modifications are collapsed.

As described by Bevan *et al.* (1998), the above proteins were grouped by functional category. They belonged to the categories of Cytoskeleton, Metabolism, Signal transduction, Transport, Proliferation/Apoptosis/Differentiation, Stress response, DNA repair, Transcription regulation, Cell adhesion and Immune response, respectively. An important fraction of proteins was associated with the cytoskeleton and were involved in metabolism. Protein-protein interaction maps of the differentially expressed protein spots in artery of obstructive jaundice patients (Figure 3), with an average node degree of 4.75, an average local clustering coefficient of 0.405, an expected number of edges of 7, and with a PPI enrichment p-value of less than 1.0e^-16^. The enrichment results indicates that the proteins are at least partially biologically connected, as a group.

## Discussion 

Previous studies have reported vascular hypo-reactivity in OJ cases as well as reduced aortic response to vasoactive products; further animal studies revealed that this effect was attributed to deregulated expression of receptors or related proteins in vascular smooth muscle cells and endothelial cells. To confirm this clinical phenomenon, we compared vascular reactivity to norepinephrine in obstructive jaundice and non-obstructive jaundice patients by non-invasive and invasive monitoring methods. Then, differential protein expression was assessed in blood vessels of these two groups by proteomic analysis. 

In the current clinical trial, we identified dose dependent hemodynamic changes after norepinephrine infusion, and changes in HR, CVP, CO and CI showed no marked differences between the OJ and control groups, reflecting similar cardiac contractility and blood volume in both groups. What’s more, differential responses of MAP, SVR and SVRI to the vasoconstrictor were found in both groups, confirming vascular hypo-reactivity in the artery and aorta of jaundice patients to certain vasoactive agents. Vascular hypo-reactivity in OJ has long been reported clinically, however, this seems to be the first prospective research to analyses the circulatory functions and their reactions toward norepinephrine in these patients.

By using 2-DE separation of proteins from whole extracts and followed by mass spectrometry, we compared proteomic profiles between artery samples from jaundice and non-jaundice patients, and revealed a total of 24 differentially expressed proteins. 2-DE technology provides protein separation by its hydrophobicity, which could identify the first axis of isoelectric point, and thus provides good mixture simplification, with the fractionated proteins intact and in the liquid phase for easy isolation and analysis with other analytical techniques such as MS, and Western blot analysis. Previous studies have already proved that 2-DE chromatography analysis performed well in the identification of novel proteins with high confidence in human amniotic fluid (Michaels *et al.*, 2007; Bujold *et al.*, 2008). We further investigated seven abnormally expressed proteins which were closely related to vascular hypo-reactivity of jaundice patients, independently confirming their expression trends by immunoblot. 

Functional annotation of proteins with differential expression in jaundice patients revealed approximately 33% were associated with the cytoskeleton, while 17% were involved in metabolism. These findings validate our hypothesis, since lower reactivity occurs during cytoskeleton structure modifications in VSMCs and/or endothelial cells (Akata, 2007), as well as changes in cellular metabolism. Actin cytoskeleton signaling emerging from the above proteomics data depicting proteins with differential expression in OJ, seemed to have an important function in vascular hypo-reactivity in jaundice patients (Schildmeyer *et al.*, 2000). These findings suggested that vascular hypo-reactivity in OJ patients is probably due to an impairment of actin-mediated muscle contractile activity. The contractile system of vascular smooth muscle comprises thin and thick filaments made of actin and myosin, respectively. Actin polymerization and cytoskeletal dynamics regulate force development in vascular smooth muscle, which results in blood pressure and blood flow regulation. In α-actin deficient mice, vascular contractility and blood pressure homeostasis are impaired, with compromised vascular contractility, decreased blood pressure, and reduced blood flow after α-actin gene transcription in the smooth muscle is inactivated (Tang and Anfinogenova, 2008). 

As shown above, actin was impaired in the artery of OJ patients; meanwhile, several actin-binding proteins were also downregulated in OJ patients. Tropomyosin is known to bind actin filaments and interact with multiple actins binding proteins, playing significant roles in muscle contraction and thin-filament assembly in both muscle and non-muscle cells. Greenberg MJ demonstrated that the intra-strand reinforcement by smooth muscle tropomyosin increases persistence length by 1.5-fold (Greenberg *et al*., 2010). The conserved periodic sites of tropomyosin are also important in regulating actin-myosin interaction that produces force generation between actin filaments, leading to contractions. Hence, beta-tropomyosin upregulation in the present study in blood vessels may probably lead to vascular hypo-reactivity in OJ patients. According to the above results, annexin A2 and A5 were also upregulated in OJ. Annexin represents a calcium-dependent phospholipid-binding protein that contributes to the exocytosis of intracellular proteins (Burger *et al*., 1996), and modulates several cell events, including motility, membrane-associated protein complex binding to the actin cytoskeleton, endocytosis and fibrinolysis (Bharadwaj *et al.*, 2013). Recombining actomyosin, annexins, and caveolar fats in presence of calcium ions generates a well-arranged precipitate; such arrangement ensures efficient transmission of contractile activity in smooth muscle cells (Babiychuk *et al.*, 1999). Another increased protein was gelsolin, a calcium-regulated actin filament severing and nucleating protein found in many cell types which is involved in various cellular processes, including regulation of actin dynamics, cell motility, control of apoptosis and cellular signal transduction. Knockdown of the gelsolin-like protein CapG in pulmonary arterial smooth muscle cells (PASMCs) causes reduced proliferation, enhanced apoptosis and cell cycle arrest, while its downregulation attenuates pulmonary hypertension. This indicates CapG contributes to pulmonary vascular remodeling (Xu *et al.*, 2016). CapG promotes monomer assembly into filaments and also severs existing filaments. Further studies to elucidate the physiological role of gelsolin are under way in our laboratory. Of note, stress resistance and actin organization related heat shock protein (HSP) were also overexpressed in OJ patients. HSP regulates the actin cytoskeleton in differentiated smooth muscle cells due to elevated amounts of this protein as well as co-localization with contractile proteins (Bitar, 2002). Stretch causes remarkable structural disruption of the actin cytoskeleton and decreases the F/G-actin ratio in bladder smooth muscle cells (BSMCs) *in vitro* with HSP27 over-expression and knock-down, indicating an important role for HSP27 in bladder smooth muscle contraction (Smoyer et al., 1996). In smooth muscle samples after permeabilization, agonist-associated contraction is suppressed by anti-HSP27 antibodies, reducing HSP27 phosphorylation inhibits vasoconstriction caused by angiotensin II (Webb, 2019). In the present study, HSP27 was decreased in OJ patients as assessed by proteomic analysis and further confirmed by Western blot. The last upregulated actin binding protein in OJ was cofilin-1. Actin depolymerizing factors (ADFs)/cofilins are widely found in eukaryotes, with diverse functions based on highly complex associations with both monomeric and filamentous actins (Remedios *et al.*, 2003). Cofilin binds to F-actin and is involved in the regulation of actin cytoskeleton dynamics and cell morphology (Ostrowska and Moraczewska, 2017). Transgelin is an actin-binding protein but was lowly expressed in OJ patients. This protein is also known as smooth muscle 22 α (SM22α). It is specifically found in the contractile smooth muscle of mammalians alongside additional differentiation markers such as α-actin, calponin and smooth muscle myosin. In SM22a(-/-) mice, contraction of aortic rings in response to angiotensin II (Ang II) is significantly decreased *in vitro*, and extracellular signal-regulated kinase 1/2 (ERK1/2) phosphorylated levels are reduced, suggesting SM22α promotes AngII-associated contraction via maintenance of the ERK1/2 pathway (Webb, 2019), meanwhile, actin amounts are reduced by 10-25% in vessels from transgelin knockout mice (Schildmeyer *et al.*, 2000). In OJ patients, whether transgelin downregulation occurs through actin or actin upregulation is through transgelin requires further investigation.

Besides actin binding proteins, proteins related to actin regulation were decreased in the artery of OJ patients. LIM and SH3 domain protein 1 (LASP) has a critical function in regulating the dynamic activities of the actin cytoskeleton. Agonist-associated LASP1 phosphorylation alterations might also modulate actin-related ion transport in parietal cells as well as other secretory epithelial cells expressing high F-actin levels. This novel actin-binding protein could control a pathway contributing to cell cytoskeleton organization (Schreiber *et al.*, 1998). Adapter proteins, such as Sorbin and SH3 domain-containing protein 2 (SORBS), play roles in signaling complex assembly, linking ABL kinases and the actin cytoskeleton. SORBS2 is an adapter protein that functions in cytoskeletal organization, cell adhesion and signaling pathways.

Other non-actin related proteins significantly altered in OJ included vimentin, one of the main structural constituents of IFs in multiple cells, plays a critical role in essential cell functions, including contractility, migration, stiffness, stiffening and division. Vimentin-deficient cells exhibit reduced contractility, indicating vimentin IFs are essential in preserving mechanical interactions among cells (Wang and Stamenovic, 2002). Considering the apparent upregulation of this protein, we also propose that it may have a critical function in vascular hypo-reactivity in OJ, which deserves further animal experiments.

## Conclusions

The present study confirmed vascular hypo-reactivity to norepinephrine in OJ patients, and several deregulated proteins were further determined by proteomic analysis. The present findings suggested that several actin or actin-related proteins in the actin cytoskeleton signaling pathway may probably be largely involved in vascular hypo-reactivity in OJ patients. Although with several limitations, for instance, smooth muscle cells and epithelial cells of the artery were not separated in this analysis, we believe that this study provides valuable evidence and directions for further investigations. 
